# Metabolic Erosion Primarily Through Mutation Accumulation, and Not Tradeoffs, Drives Limited Evolution of Substrate Specificity in *Escherichia coli*

**DOI:** 10.1371/journal.pbio.1001789

**Published:** 2014-02-18

**Authors:** Nicholas Leiby, Christopher J. Marx

**Affiliations:** 1 Organismic and Evolutionary Biology, Harvard University, Cambridge, Massachusetts, United States of America; 2 Systems Biology Graduate Program, Harvard University, Cambridge, Massachusetts, United States of America; 3 Faculty of Arts and Sciences Center for Systems Biology, Harvard University, Cambridge, Massachusetts, United States of America; Yale University, United States of America

## Abstract

Evolutionary adaptation to a constant environment is often accompanied by specialization and a reduction of fitness in other environments. We assayed the ability of the Lenski *Escherichia coli* populations to grow on a range of carbon sources after 50,000 generations of adaptation on glucose. Using direct measurements of growth rates, we demonstrated that declines in performance were much less widespread than suggested by previous results from Biolog assays of cellular respiration. Surprisingly, there were many performance increases on a variety of substrates. In addition to the now famous example of citrate, we observed several other novel gains of function for organic acids that the ancestral strain only marginally utilized. Quantitative growth data also showed that strains with a higher mutation rate exhibited significantly more declines, suggesting that most metabolic erosion was driven by mutation accumulation and not by physiological tradeoffs. These reductions in growth by mutator strains were ameliorated by growth at lower temperature, consistent with the hypothesis that this metabolic erosion is largely caused by destabilizing mutations to the associated enzymes. We further hypothesized that reductions in growth rate would be greatest for substrates used most differently from glucose, and we used flux balance analysis to formulate this question quantitatively. To our surprise, we found no significant relationship between decreases in growth and dissimilarity to glucose metabolism. Taken as a whole, these data suggest that in a single resource environment, specialization does not mainly result as an inevitable consequence of adaptive tradeoffs, but rather due to the gradual accumulation of disabling mutations in unused portions of the genome.

## Abstract 2

During long-term evolution of *Escherichia coli*, nutrient specialization is primarily driven by the accumulation of neutral mutations, rather than by tradeoffs, and can also be accompanied by general catabolic improvements.

## Author Summary

Adaptation to a single constant environment is commonly expected to result in decreased performance in alternative conditions, or specialization. It has been proposed that, rather than occurring through the neutral accumulation of mutations in unused alternative pathways, this happens because loss of these pathways enhances fitness in the constant environment via “tradeoffs.” We examined growth rates across a variety of nutrients for 12 independent lineages of *Escherichia coli* that had evolved in the laboratory for decades in a glucose-containing medium. Surprisingly, after 20,000 generations there were actually widespread improvements in the use of alternative nutrients, rather than the expected declines. After 50,000 generations, however, we find that this trend reversed for those populations that evolved a much higher mutation rate. This indicates that high mutation rate, and not adaptive tradeoffs per se (as had been previously proposed), is the primary driver of specialization. These results caution against general assumptions about the importance of adaptive tradeoffs during evolution, and emphasize the key role that newly evolved changes in mutation rate can play in promoting niche specialization.

## Introduction

Evolving populations face the fundamental dilemma that there is no single phenotype that is optimal in all environments. When an evolving population occupies the same selective environment for an extended period of time, no advantage is realized by maintaining fitness on resources it no longer encounters. Adaptation to a selective environment can result in correlated responses in alternative environments. Although these can be synergistic improvements, a response that decreases fitness in other environments is known as specialization. This prevents the rise of “Darwinian demons”: single supergenotypes that are optimized across all conditions [1]. It is critical to understand the origin of specialization because it underlies the origin and maintenance of diversity—it is why “the jack of all trades is a master of none” [2].

Specialization can result from either selective or neutral processes. Antagonistic pleiotropy describes when natural selection favors changes that are beneficial in the current environment but reduce function in other environments. Alternatively, specialization may result from mutations that decrease fitness in alternative environments that are neutral in the selective environment. These mutations have the potential to either drift or hitchhike to fixation via “mutation accumulation.” As neutral mutations accrue in proportion to the mutation rate, the clearest evidence of mutation accumulation can come from excess specialization in mutator lineages, which contain defects in mutational repair that can elevate mutation rates ∼100-fold [3]. In contrast, where specialization is rapid and occurs in parallel across lineages, a pattern commonly seen for adaptation itself, this has been cited as support of selection-driven antagonistic pleiotropy [4].

The experimental evolution of 12 populations of *Escherichia coli* grown for thousands of generations on a single substrate has been used to distinguish whether selective or neutral processes drive metabolic specialization [4]. The populations were part of the Lenski Long-Term Evolution Experiment (LTEE) [5], in which wild-type *E. coli* B have been diluted 1∶100 daily and regrown in well-mixed medium containing glucose as the sole usable carbon source. After 20,000 generations (20k), competitive fitness on glucose had increased by ∼70%. However, respiration assays in static 96-well Biolog plates (Hayward, CA) [6] suggested dramatic decreases in metabolic performance on alternative substrates. These declines occurred rapidly and in parallel across populations, coincident with the largest gains in fitness. This was suggested to indicate selection-driven antagonistic pleiotropy as the main mechanism of specialization. Furthermore, because there was only a weak, nonsignificant excess of declines by the populations which had become mutators earlier in the experiment [3], this suggested neutral mutation accumulation played little role, if any, in glucose specialization by 20k.

In this study we have readdressed the basis of specialization in the LTEE populations, motivated by our discovery that the growth rates of isolates in well-mixed media are poorly captured by assays of cellular respiration in static, proprietary media. Given this surprising finding, we analyzed the selected (i.e., glucose) and correlated responses of isolates from both 20k and 50,000 generations (50k) from four perspectives:

- Generality and parallelism of specialization—Is there an overall pattern of decreased growth performance on alternative substrates? Is the pace and pattern of declines in function consistent with antagonistic pleiotropy?
- Novel gains of function—Besides the prior example of evolution to catabolize citrate present in the medium [7], are there any other substrates used by evolved isolates that are not utilized by the ancestors?
- Role of elevated mutation rate driving mutation accumulation—Will the 30,000 generations that have passed since 20k have lead to sufficient mutation accumulation for the mutators to exhibit significantly greater decreases in growth than nonmutators?
- Predictability of catabolic declines—Can we predict which substrates would be metabolized less effectively based upon the similarity of their use to glucose? Is there a pattern to this specialization that might suggest a common biophysical basis behind them for the mutator lineages?

## Results

### Respiration Assays Indicated Broad Declines in Function Across Substrates, Including Those Where Adaptation Occurred

As a first step to readdressing specialization in the LTEE populations, we sought to replicate the Biolog respiration results at 20k presented by Cooper and Lenski [4], as well as extend this analysis to the populations at 5ok. Despite changes in the Biolog assay itself, since the previous study, we recovered a similar pattern for the panel of substrates (Figure S1).

**Figure 1:**
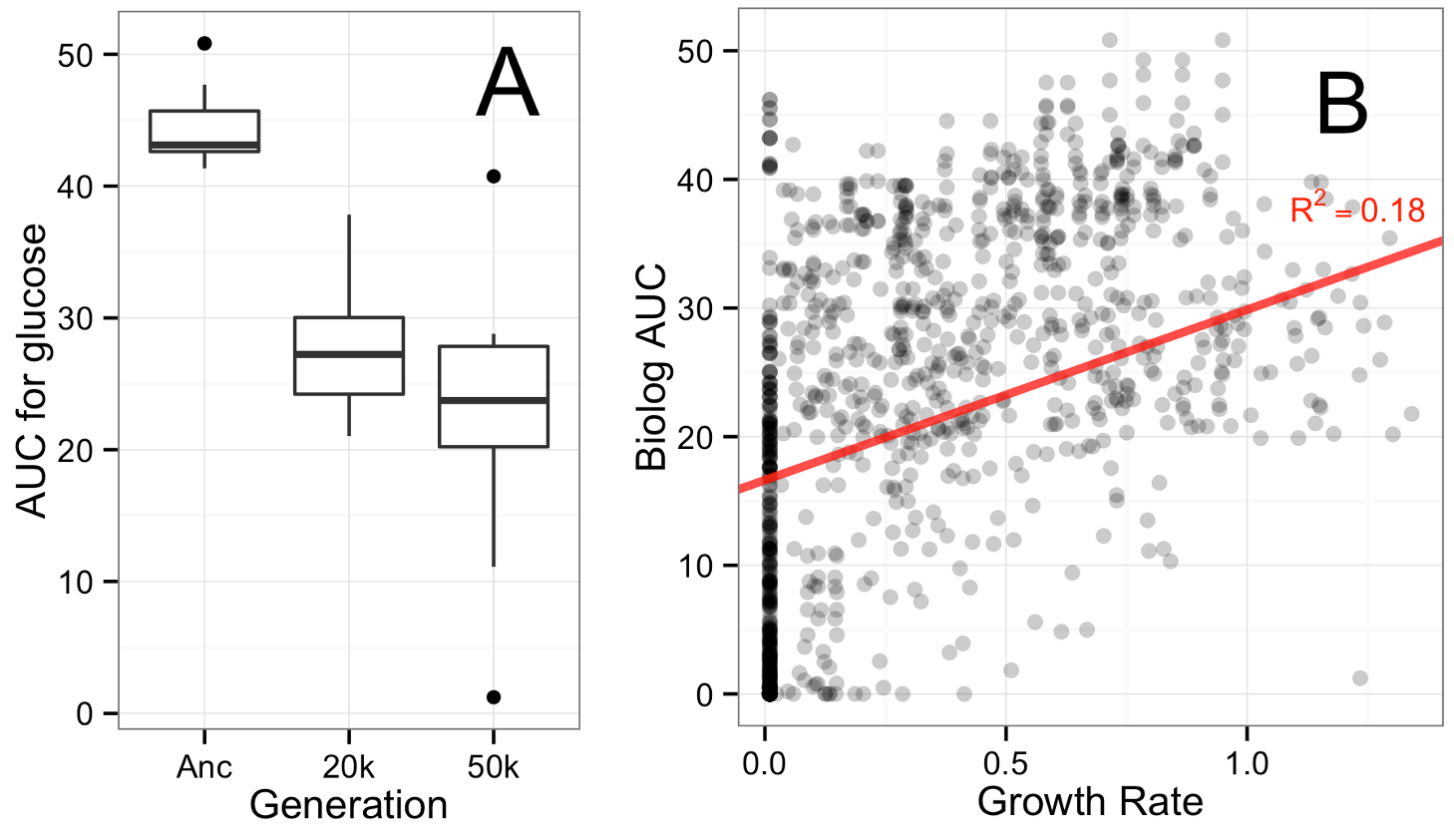
Biolog measurements are a poor proxy for growth performance. (A) Biolog AUC as measured for the D-glucose on Biolog plates. The evolved strains have a lower AUC value than the ancestor on glucose, the carbon source available during evolution (*p*<0.0001, Welch's two sample *t* test). The mean AUC for the 20k and 50k isolates on glucose are not statistically different. (B) Scatter plot showing the measurement of function as Biolog AUC versus growth rate on all substrates, for all strains at 20k and 50k generations as well as the ancestors. The regression shown is for substrates after removal of categorical disagreements (growth without respiration or respiration without growth, 167/702 in total).

The validity of Biolog assays as a proxy for strain improvement came into question after finding decreases for the very substrates on which selection occurred (Figure 1A). Despite abundant evidence of improvement from competitive fitness assays [4] and growth rates [8], respiration on glucose consistently decreased over the course of the experiment. Furthermore, although one lineage in the A-3 population evolved to utilize as a carbon source the citrate included in Davis Minimal (DM) medium [7], it produced a statistically indistinguishable respiration value from the other Cit^−^ isolates at 50k (Figure S2).

### Growth Rates Reveal a Balance of Decreased and Increased Performance Across Substrates

Given that the selective substrates with known fitness improvement had decreased cellular respiration, we turned to direct measurements of growth rates across a wide panel of substrates using a robotic growth analysis system [9],[10]. By measuring growth rate we capture the demographic metric best correlated with competitive fitness in the evolutionary environment [11]. As the LTEE was performed in shaken, fully aerated flasks, these well-mixed 48-well plates were a closer match to the evolutionary environment than unshaken 96-well plates, as unshaken plates commonly exhibit subexponential growth due to oxygen limitation [10]. Although Biolog uses a proprietary minimal media, for the growth rate measurements we used the same DM media as the LTEE experiment. We omitted citrate, however, as this choice allowed us to include the Cit^+^ A-3 population in our analysis. We chose carbon sources based upon the substrates for which significant, parallel decreases were previously observed via respiration assays [4], as well as citrate and several sugars on which growth tradeoffs were previously measured after 2,000 generations [12].

Comparing growth rates to respiration data, it becomes evident that the latter is not an accurate assay for growth (Figure 1B). There were many cases where respiration occurred without growth, as 156 out of the 702 strain/substrate combinations measured did not permit growth but did have measurable respiration—a known feature of Biolog assays [6]. Even after removing these categorical disagreements, and a smaller number of instances of growth without respiration (11 strain/substrate combinations), Biolog respiration values were a poor predictor of growth rate [R^2^ = 0.18, linear regression *F* test(1,499) = 108.8].

**Figure 2:**
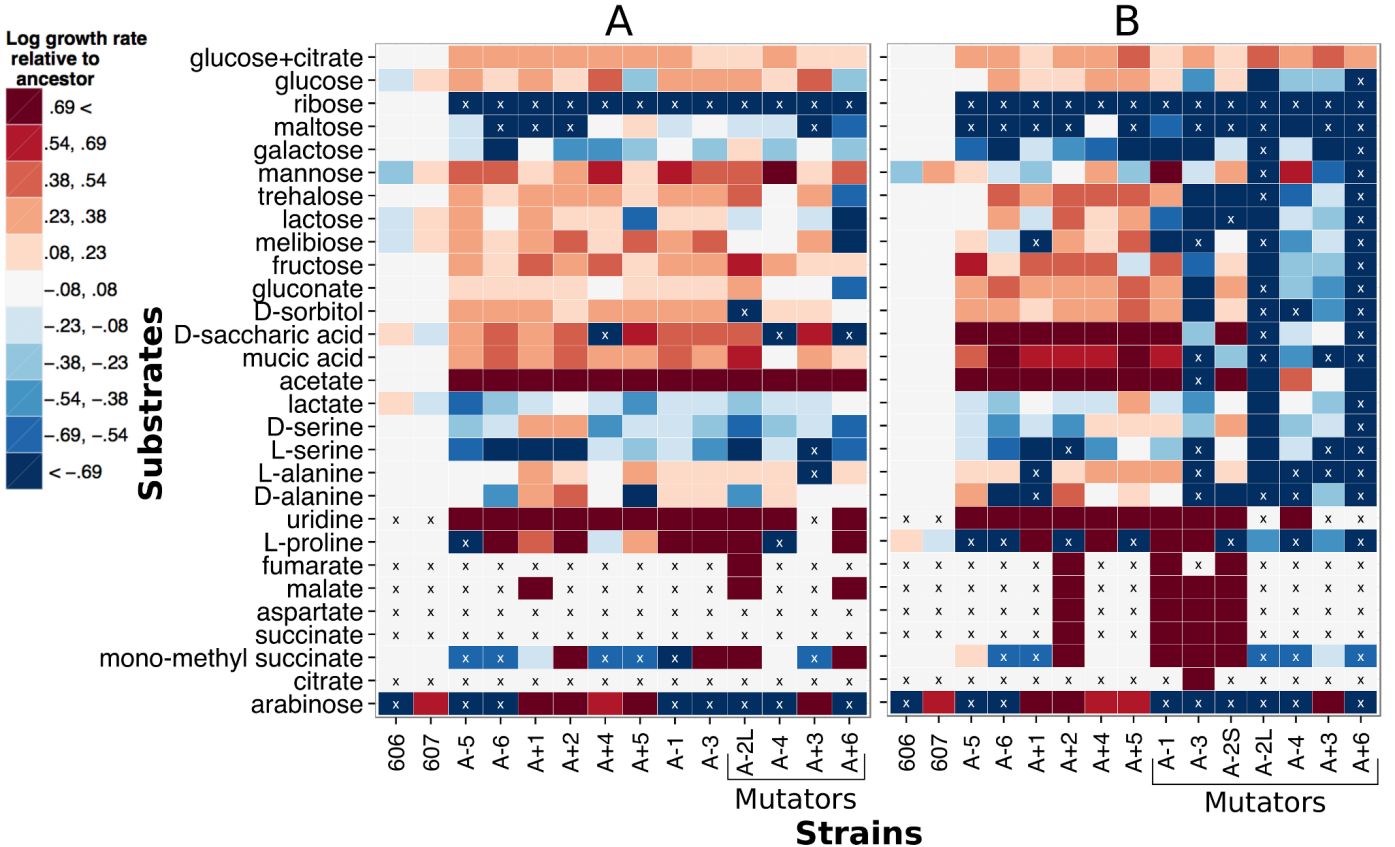
Relative growth rates across a variety of growth substrates for evolved strains from 20k (A) or 50k generations (B). Heatmaps indicate the log ratio of growth rates relative to the average of the two ancestors on that carbon source. White indicates a growth rate equal to that of the ancestor average, red faster, and blue slower. The growth rates are plotted on a log scale with the limits of the color range set for twice as fast and half as fast as the ancestor average. An “x” in a box indicates that no growth was observed for that combination of strain and substrate over 48 h. Strains that were mutators by that time point are indicated.

Growth rate data across substrates revealed a surprising degree of correlated gains in performance (Figure 2). Indeed, at 20k, there were actually more correlated increases in rate than decreases (165 versus 99, respectively, *p*<0.0001 for binomial two-sided test with null of random gains and losses). By 50k, the picture had reversed, now with more decreases than increases (167 versus 119, *p* = 0.005, binomial two-sided test).

### Gains of Function on Alternate Carbon Sources

In addition to many quantitative improvements of growth rates, there were several examples where isolates acquired the ability to grow on substrates that the ancestral strain could not utilize over the 48 h time-course of the growth experiments. Only one such example was previously known: the aforementioned gain of citrate utilization by the A-3 population [7]. We found that this strain also gained the ability to grow within 48 h on three C_4_-dicarboxylate tricarboxylic acid cycle intermediates (succinate, aspartate, and malate). Three other 50k isolates from different replicate populations gained the ability to use this same set of three C4 dicarboxylate intermediates, as well as fumarate.

### Mutator Strains Suffered Greater Declines in Growth on Alternative Substrates

We compared mutators to nonmutators to ask whether mutation accumulation contributed to the observed decreases in growth (Figure 2). At 20k generations, despite increasing in growth rate more than decreasing (47 versus 41 cases), the mutators were marginally worse, on average, than nonmutators (*p* = 0.03, Pearson's chi-squared test comparing proportion of growth rate reductions). By 50k there was a stark pattern of mutators declining in catabolic ability compared to nonmutators (*p*<0.0001, Pearson's chi-squared test). This can be seen in the large block of blue (decreases in rate) for five of the mutators. Nonmutators at 50k still increased in growth rate more often than they decreased (80 versus 52 cases, *p* = 0.018 binomial two-sided test). Because some strains are known to be affected by the absence of citrate even though they cannot use it as a carbon source [13], we also tested the growth rate of the 50k strains on alternative substrates supplemented with 1.7 mM sodium citrate, as in LTEE growth media. Although the reductions in growth rate relative to the ancestor were ameliorated in some cases by the addition of citrate, the mutator strains still suffered significantly more growth rate decreases than the nonmutators (*p* = 0.003, Pearson's chi-squared test).

### Parallelism of Metabolic Decreases

Given the trend of both increased and decreased growth rate on alternative carbon sources, we assessed the degree of parallelism with which metabolic erosion occurred. We segmented the data by substrate and asked how many evolved strains decreased in growth rate or cellular respiration on each substrate. We took as a null expectation that decreases in metabolic function are equally as likely as increases, and plotted the observed pattern against this null distribution (Figure S3).

In no case do our observations of metabolic decreases closely match the null distribution. As previously, cellular respiration was reduced for nearly all strains on all substrates. The observed average number of strains with reduced respiration on a substrate was 11.3 at 20k and 12.8 at 50k, 5.3 out of 12 and 6.3 out of 13 more strains than would be expected given the null distribution (*p*<0.0001, binomial two-tailed exact test). For growth rate, at 20k on average 5.3 strains reduced in growth rate on each substrate, in fact 0.7 fewer strains than expected given the null distribution (*p* = 0.01). However, this average somewhat masks the bimodal pattern seen in the distribution, with some substrates showing nearly no strains reducing growth rate and others nearly all. At 50k, an average of 8.3 strains lost function on each substrate, 1.8 more than expected (*p*<0.0001). Clearly there is some parallelism in decreases in growth rate, but it is worth emphasizing that the substrates used in this study were those for which widespread, parallel losses in cellular respiration were previously observed.

### Metabolic Similarity Between Substrates Was a Poor Predictor of Correlated Evolved Responses

We asked whether the correlated changes in performance on alternative substrates could be predicted based on the similarity of the catabolic network for growth on that compound compared to that for glucose. There are two rationales that would support this hypothesis. First, there are more loss-of-function mutations available for a nonglucose substrate if it uses many unique enzymes, and we might expect to see metabolic specialization scale with mutational target size under mutation accumulation. These mutations may either simply be unguarded by purifying selection, or perhaps even selectively advantageous to lose. Second, the balance and direction of flux through various pathways will lead to a different optimal allocation of enzymes to balance the needs of catalysis versus expression costs. As such, antagonistic and synergistic pleiotropy suggest that highly overlapping metabolic flux patterns might be expected to suffer fewer declines, or possibly even synergistic gains, relative to a very differently used substrate. A rough approximation of the similarity between different substrates is to simply group them as sugars or “nonsugars” that require gluconeogenesis for anabolism. To frame this more quantitatively, however, we also used genome-scale metabolic models to make predictions about specialization.

In order to approximate internal metabolic states, we used flux balance analysis (FBA) to generate predicted flux patterns for each compound [14]. This approach generates a vector of internal flux values that describes the relative flow through every reaction in a cell were it to optimize biomass production per substrate molecule. Although selection in batch culture largely acts upon rate, biomass production per unit substrate has been shown to effectively capture the growth of the LTEE ancestor on glucose, and 50k evolved strains deviated only slightly from this pattern [15]. We therefore compared FBA-derived flux vectors using a number of metrics to determine their degree of dissimilarity to the flux vector for glucose (see Materials and Methods).

**Figure 3:**
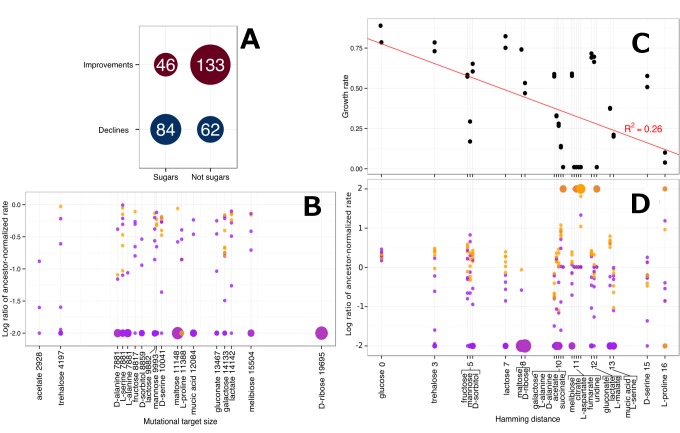
Substrate dissimilarity does not predict metabolic erosion. (A) A simple categorization of substrates as sugars and nonsugars finds that the correlation between relatedness to glucose and evolved metabolic changes is the opposite from what is hypothesized. (B) The FBA-predicted mutational target size does not correlate with decreases in growth rate. (C) Hamming distance between FBA-generated flux vectors for carbon sources partially predicts ancestral growth rate. Black dots indicate the growth rate of the two ancestral strains. A total of 268 reactions were predicted as necessary for optimal metabolism on glucose. (D) Hamming distance between a substrate and glucose does not correlate with increases or decreases in growth rate. The *y* axis is the log of the ratio of growth rate relative to the ancestor, with all ratios greater or less than *e* ^2^ binned at the axis limit. For (C–D), purple dots are mutator strains, and orange dots are nonmutators. Larger dots at the axis extrema indicate more overlapping points, and the shading between purple and orange indicates the different proportions of mutators and nonmutators at that limit. For (B–D), substrates with the same *x* axis values were plotted with a slight offset, and the true value is listed in the axis label.

We first tested whether evolved decreases in growth rate scaled with mutational target size. There is no expected behavior under this hypothesis for increases in growth rate, so we limited our analysis to combinations of strains and substrates for which growth rate had decreased. By identifying the reactions necessary for optimal growth on alternative substrates that are not necessary for growth on glucose, and determining the number of coding nucleotides necessary for those reactions, we were able to approximate the number of available mutations that would decrease growth rate on a substrate. Contrary to our hypothesis, we found no significant relationship between mutational target size and reduction in growth rate [*p* = 0.15, linear regression *F* test(1, 146) = 2.1] (Figure 3B).

Our hypothesis also suggests that substrates used more similarly to glucose would permit more rapid growth. Starting with the simple categorization of substrates as sugars and nonsugars, we found no correlation between these groupings and changes in growth rate (Figure 3A). Indeed, there were many reductions in growth rate for sugars other than glucose. To frame this hypothesis in a more quantitative way, we compared the Hamming distance between the vector of predicted fluxes for an alternative compound and that of glucose. Contrary to our hypothesis, we found no significant relationship between metabolic similarity to glucose and correlated responses [*p* = 0.26, linear regression *F* test(1, 323) = 1.27], and any relationship measured was in fact in the opposite direction as predicted (Figure 3D). As a confirmation that Hamming distance between flux vectors for alternative substrates is biologically relevant, we found that it was a significant predictor of some of the variance in the relative growth rate of the ancestor [*p* = 0.0001, R^2^ = 0.26, linear regression *F* test(1, 48) = 17.2] (Figure 3C). Alternative metrics to Hamming distance performed similarly poorly in predicting patterns of tradeoffs (Table S4). These data suggest that the similarity of overall flux patterns is a surprisingly poor predictor about which substrates would experience correlated increases or decreases in performance.

### Growth for Mutator Strain Was More Sensitive to Temperature Than for Nonmutators

Linking the mutator-driven metabolic specialization to their vastly elevated mutation rate itself, we hypothesized that their abundance of amino acid substitutions may generate trends indicative of the types of effects they had upon their gene products. The mutator lineages acquired mutations with a rate up to 0.06 per generation [16]. For the A-1 lineage, by 40,000 generations there were 627 SNPs, 599 of which were in coding regions, and 513 of those were nonsynonymous [17]. This is a tremendous load of amino acid substitutions, which are viewed as likely to be deleterious due to destabilizing proteins [18]–[20]. We therefore hypothesized that, if protein destabilization was a dominant factor affecting growth at 37°C, we could predictably ameliorate these defects by lowering the growth temperature. Nonmutators will have a small number of such mutations, but the ∼100-fold greater rate of such mutations in the mutator genomes should make growth more temperature sensitive than for nonmutators.

**Figure 4:**
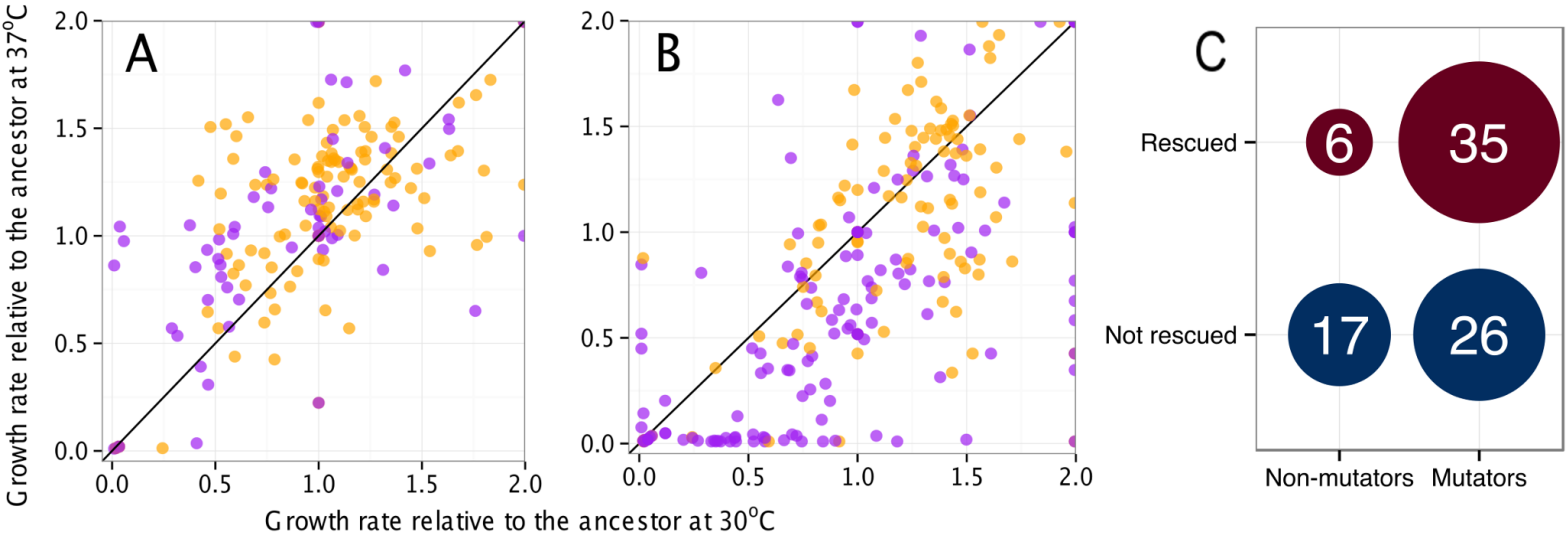
Temperature dependence of growth rate on alternative substrates. For all strain/substrate measurements, we determined the relative change in growth rate by changing temperature from 37°C to 30°C. For (A–B), purple dots are mutator strains; orange dots nonmutators. Points that fall outside of the plot range are plotted at the edge of the graph. (A) Effect of temperature change on 20k isolates. (B) Effect on 50k isolates. (C) For 50k isolates, the number of mutators and nonmutators that were rescued from no growth at 37°C to growth at 30°C.

Growth rate data support the hypothesis that mutators have general temperature-sensitivity. For 50k isolates, growth rate relative to the ancestor at 30°C was higher than at 37°C in 98 cases, compared to only 37 cases where it was reduced relative to the ancestor (*p*<0.0001, binomial two-tailed exact test) (Figure 4B). That is, despite the fact that these strains have adapted for 50,000 generations at 37°C, the ratio of their growth rate to that of the ancestor is higher at the foreign 30°C than their native temperature. This general improvement at the lower temperature was not present for nonmutators (56 improved relative to the ancestor by moving to 30°C, 57 worse—*p* = 0.99, binomial two-tailed exact test), and the difference between mutators and nonmutators was significant (*p* = 0.0002, Pearson's chi-squared test). Furthermore, in the cases where evolved 50k isolates completely lost the ability to grow on substrates, when grown at 30°C these losses were ameliorated more than half the time for mutators (35 of 61), significantly more than for nonmutators (6 out of 23, *p* = 0.01, Pearson's chi-squared test) (Figure 4C).

An alternative hypothesis for the elevated temperature sensitivity of mutators is that the phenotype is directly caused by the mutation in mismatch repair rather than the accumulation of destabilizing mutations that it caused. For the 20k isolates, in most cases growth was better relative to the ancestor at the native 37°C than at 30°C (119 versus 75, *p* = 0.002, binomial two-tailed exact test) (Figure 4A). There was no significant difference at 20k between this pattern for mutators and nonmutators (*p* = 0.20, Pearson's chi-squared test), and no significant difference in the number of rescues from complete loss of growth (*p* = 0.31, Pearson's chi-squared test), ruling out that mutator status itself generates temperature sensitivity.

## Discussion

Replicate experimentally evolved populations such as the LTEE are ideal for studying the processes leading to specialization. Here we directly measured growth rates in well-mixed conditions rather than cellular respiration in static media. This seemingly minor choice generated qualitatively different results, leading to the opposite conclusions from those previously made about the mechanisms and patterns of specialization during tens of thousands of generations of growth on a single substrate.

The earlier report [4] of widespread, parallel tradeoffs after extended growth on a single compound fit comfortably with the general notion that unused capacities will tend to degrade after an extended period of disuse. However, the suitability of cellular respiration assays for growth performance was seriously challenged by our data. There were many cases of “false positives,” with respiration on substrates that do not support growth, and a weak predictive ability for growth even after removing these. The parallel decreases in function observed across compounds in the respiration assay seem to be due to cultures becoming worse at reducing the dye in the Biolog assay environment. This generic effect was strong enough to mask both adaptation on glucose and the novel gain of citrate use in the A-3 50k isolate.

### Little Parallelism in Metabolic Erosion

Growth rates indicated little evidence in support of widespread antagonistic pleiotropy, with more increases than decreases in growth on alternative compounds through 20k, and very few parallel declines. There were individual counterexamples observed, such as the previously characterized universal loss of ribose utilization early in adaptation [21], and the tendency for reduced or loss of growth on maltose [22]–[24]. As such, it is clear that examples of antagonistic pleiotropy do exist in the data. However, relatively few other substrates showed this pattern at 20k or 50k, despite the fact that these substrates were those where parallel reductions in respiration were observed. Because selection drives antagonistic pleiotropy, it is commonly expected that the early period of rapid adaptation would coincide with the most tradeoffs in alternative environments, and that the frequent parallelism in the targets among early beneficial mutations would drive parallel losses [4]. Given these criteria, the growth data do not support antagonistic pleiotropy as the primary driver of specialization.

There are three implicit assumptions about antagonistic pleiotropy, however, that if not met alter the expectations for specialization driven by selection. First, if different beneficial mutations occur across lineages, they will not necessarily lead to the same pleiotropic tradeoffs. As of 20k, out of the 14 genes screened in all of the populations, there were three genes with mutations in all populations and two more in a majority. The other screened genes had mutations in a minority or none of the other populations, suggesting that a variety of different beneficial mutations occurred across lineages [17]. Second, beneficial mutations in the same target may have differing pleiotropic effects in different lineages due to other mutations present. This “epistatic pleiotropy” [25] has been found to be common in multiple model systems [26]–[29]. Third, the early large-effect beneficial mutations may or may not be responsible for greater pleiotropic effects than later, smaller effect mutations. Yeast morphological pleiotropy scaled with fitness, for example, but the correlation explained only 17% of the variation [30]. The first and second scenarios above—distinct mutations or epistatic pleiotropy—would lead to a scenario whereby parallel metabolic declines are no longer necessarily expected from antagonistic pleiotropy. The third scenario—pleiotropy not scaling with selective effect—would mean the temporal dynamics of fitness gain in the selective conditions and the rate of performance losses in alternative environments need not be tied.

These caveats underscore our limited ability to make conclusions about the role of antagonistic pleiotropy in the observed metabolic declines. The only sure determinant of whether a correlated change is the result of pleiotropy or neutral mutation is to genetically manipulate the strains to isolate the effect of individual mutations. This suggests that future experiments, for example, test early and parallel mutations previously screened for epistatic effects [31] for pleiotropy. Ultimately, as we discuss below, the key determinant of the role of mutation accumulation is whether metabolic specialization was substantially affected by mutation rate.

### Increases in Growth Rate on Carbon Sources Other Than Glucose

Rather than a general pattern of metabolic specialization, these data revealed an unexpected extent of correlated improvements in growth on alternative compounds. Why would *E. coli* maintain or improve performance on substrates that had not been supplied for decades? There are three general classes of explanations, two of which mirror the processes considered for pleiotropic tradeoffs.

The first explanation for the correlated improvements, and undoubtedly the least likely, would be neutral performance gains through mutations that had no selective consequence in glucose: the beneficial analog of mutation accumulation. If this were the case, mutators might have more increases in rate than nonmutators, and more improvements would have occurred by 50k than 20k, which is the opposite of what was observed.

The second explanation of the correlated improvements is that the same mutations that were beneficial during growth on glucose may have led to gains in alternative environments—that is, “synergistic pleiotropy.” There are known examples of this occurring for early mutations in *E. coli* evolving in these conditions [12],[32], and it is a common pattern seen across organisms (for example [33]). These synergistic mutations may be generally beneficial in the laboratory environment of the LTEE and thus unrelated to carbon source metabolism. Indeed, there are examples of both adaptation to generic aspects of a selective environment, such as the trace metal formulation [34], and removal or down-regulation of costly genes or genome regions [21],[35],[36]. Synergistic pleiotropy could also result from mutations that directly improved glucose metabolism, such as mutations in the phosphotransferase-mediated uptake system that also increased growth on the other sugars imported by this system [12].

A third hypothesis for correlated gains of function on alternative compounds is that there were additional compounds besides glucose (and citrate) available from cell excretions or lysis. The serial transfer regime of the LTEE creates a scenario whereby populations use all of their glucose resources within the first few hours, and remain in stationary phase the remainder of the day. The ancestral *E. coli* excrete a small amount of acetate in this environment, and this increased on average 2-fold by 50k generations [15]. It is thus unsurprising that the strongest, most universal gain in alternative compounds by 20k was on acetate (Figure 2A). In terms of cell lysis, this has allowed one population (A-2) to maintain a long-term polymorphism for over 40,000 generations. A “large” colony lineage that grows fast on glucose but lyses substantially in stationary phase cross-feeds a “small” colony lineage that is not as fast on glucose as the larges but has specialized as a “cannibal” [37]–[39]. This results in a stable, negative frequency-dependent fitness effect between these strategies. Although an earlier study of the other populations at 20k failed to reject that fitness interactions were transitive through time [40], these competitions were performed at a 50∶50 ratio and thus may have missed interactions that occur when one partner is rare.

### Novel Gains of Function

The most remarkable correlated increases were the several examples of “novel” gains of function by evolved isolates on substrates where the ancestor failed to grow. The citrate example has been reported previously [7], but we did not expect to find other such substrates. These novel gains of function are distinct from what was seen for citrate, as over a longer duration (>100 h) the ancestral *E. coli* seem to grow to measurable density on these substrates. We are currently exploring whether these long lags represent slow physiological acclimation or the emergence of evolved genomic changes. In the case of the Cit^+^ A-3 lineage, succinate is likely excreted during citrate import [41]; thus, selection for its use is perhaps unsurprising. The fact that several other strains experienced similar gains across the same range of C4 dicarboxylic acids, and that this included the cross-feeding “small” phenotype clone from the A-2 population, appears to suggest that these compounds may be excreted, or present during stationary phase from lysed cells.

### Predictability of Correlated Responses on Alternative Carbon Sources

With the availability of whole-genome scale metabolic models for *E. coli*, we asked whether we could predict the trend of correlated responses by comparing their pattern of use to that of glucose. We proposed a generic, seemingly obvious hypothesis that the more different the metabolism of an alternative substrate was from the metabolism of glucose, the more likely it would be that populations would have decreased (or lost) their ability to use it. As described, this logic holds regardless of whether or not selection drove metabolic specialization. In order to quantify the similarity of substrates, we applied FBA to compare the predicted optimal metabolic flux states for each compound. The data, however, did not support our hypothesis: there was almost no relationship between similarity to glucose and correlated response. Recent *in silico* attempts to predict growth capability on substrates based on metabolic similarities have had some success [42], suggesting that evolution may be acting here on functions not included in the model. For example, mutations may have occurred in functions related to differential regulation that distinguish these sugars, rather than central metabolic enzymes for which their use is nearly identical. These mutations are known to have occurred in the LTEE, for example in *spoT* and *nadR* [35],[43]. These predictions are also based on the assumption that glucose is the only available carbon source. If growth on other carbon sources is under selection due to their excretion or presence after cell lysis, it may explain some of the lack of predictive power here.

### Temperature Sensitivity of Mutators Consistent with Protein Destabilizing Mutations

Although the identity of the substrates that experienced tradeoffs (or improvements) were not those we expected, we reasoned that the biophysical effects of the deluge of mutations in mutators might lead to a predictable pattern of temperature sensitivity in these strains. The genomic sequences and data available to date [16],[17] suggest mutators will have on the order of 500–2,000 nonsynonymous mutations, perhaps more. Random amino acid substitutions have been shown to be mildly deleterious in general due to destabilizing proteins [20]. To ask whether tradeoffs observed in the mutators were at least partly due to destabilizing mutations in proteins needed for alternative substrates, we tested whether mutators would be more sensitive to changes in incubation temperature than nonmutators. Consistent with this hypothesis, we found that the 50k mutators performed better relative to the ancestor at 30°C than at the 37°C temperature where they have evolved. One alternative hypothesis that was ruled out is that the mutator allele itself leads to temperature sensitivity, as the 20k mutators performed better at 37°C than at 30°C, and the changes with temperature were not distinguishable from nonmutators. Although other alternative hypotheses may explain some of the temperature sensitivity, these data are consistent with the hypothesis that neutral degradation of protein-coding sequences in these strains proceeded via partial destabilization on the way to eventual loss of function.

### Role of Elevated Mutation Rate Driving Metabolic Specialization

The comparison of mutators and nonmutators at 50k strongly suggests that neutral mutation accumulation was the primary driver of metabolic specialization. The difference in metabolic erosion allows us to distinguish the overall trend from forms of antagonistic pleiotropy that could have lacked parallelism (different mutations or epistatic pleiotropy) or that may have arisen late relative to fitness gains (if pleiotropy did not scale with selective effects). Despite a significant difference from nonmutators in the proportion of growth rate reductions, after 20,000 generations and a decade of adaptation the mutators still increased growth rate in more cases than they decreased, and only by 50k did mutators as a group have more decreases than increases. By the later time point, five of the seven mutators had decreased growth (or complete loss) for essentially every single alternative compound (except citrate and C4 dicarboxylic acids for A-3, which were under selection for this strain). Interestingly, the other two (A-1, A-2S) do not show this pattern. These counterexamples may be due to the fact that A-1 acquired its mutator status late [17], and A-2S is the cross-feeding generalist described above that adapted to grow upon lysed cell material [38].

The late appearance of metabolic erosion argues for the unparalleled utility of truly long-term experiments. A neutral process such as mutation accumulation needs time to become apparent, although hitchhiking with beneficial mutations can speed their fixation (i.e., “draft” [44],[45]). With a reduced effective population size, the window of selective effects that behave neutrally grows. As such, the effects of elevated mutation rates and mutation accumulation become apparent much more quickly with evolution regimes with small bottlenecks, such as single colonies [46],[47]. The late appearance of specialization also contrasts sharply with abundant evidence that lineages can diversify and specialize in mere tens or hundreds of generations. In addition to population size, this difference in timescale appears to correlate with the type of selective environment, and thus the evolutionary process that was responsible. Whereas the Lenski LTEE is notable as an environment with a single nutrient resource at high concentration, the cases of rapid diversification have involved spatial heterogeneity [48], rate-limiting resources in a chemostat [49], or the presence of multiple substrates simultaneously [50]. In those scenarios, selection is actively pulling on different performance features of an organism and antagonistic pleiotropy appears to dominate. The relatively slow degradation of catabolic capacity in the LTEE suggests that *E. coli* faces comparatively little tension between improving upon glucose and maintaining performance on other substrates, even those which are predicted to be utilized in a very distinct manner. Specialization in this case appears not to have been a requisite tradeoff of adaptation, but rather a result of the degradation of unneeded proteins.

### The Fate of Mutator Strains in the Long Term

Given the severity of metabolic erosion for mutators even in large laboratory populations, it is remarkable just how common mutator lineages are in nature. Mutators have been isolated at frequencies over 1% and seem to be particularly common in organisms such as pathogens [51]. The frequency of mutators in nature, despite the associated costs, may be partially explained by increased evolvability, shown in laboratory medium [52] and in mice [53].

Our results add substantially to the idea that an elevated mutation rate is an ill-fated long-term strategy even for large populations, as declines in performance in alternative environments will eliminate previously occupied parts of the niche space. Recent findings have suggested that mutators may tend to attenuate their increased mutation rate over time [16],[54],[55], perhaps to avoid the harmful effects of Muller's Ratchet. Thus both tradeoffs in alternative environments and mutation load in selective environments may contribute to the paradox that over the short term lineages often benefit from elevated mutation rates, but the long-term trend across phylogenies has been for stability in mutation rates of free-living microbes [56].

## Materials and Methods

### Strains and LTEE Conditions

*E. coli* B isolates were obtained from the LTEE [5] after 20,000 and 50,000 generations. Briefly, in the evolution experiment 12 populations of *E. coli* B were founded with either the arabinose-negative strain REL606 (populations A-1 to A-6) or the otherwise isogenic arabinose-positive derivative, REL607 (A+1 to A+6). These have been evolved since 1988 in 50 mL flasks containing 10 mL of DM media with 139 µM glucose (25 mg/L) as a carbon source. The cultures were grown at 37°C while shaking at 120 rpm, and were transferred daily via 1∶100 dilutions (∼6.64 net doublings per day).

The isolates analyzed in this experiment consisted of the ancestral lines REL606 and REL607, as well as the “A” clone frozen at 50k and 20k generations for the 12 populations. The A-2A clones at 20k and 50k were from the “large” lineage that has coexisted with a cross-feeding “small” lineage for tens of thousands of generations [57], and thus here we refer to them as A-2L. At 50k we also examined A-2C (REL11335), a “small” clone that we refer to here as A-2S. All evolved strains are listed in Table S1.

### Growth Rate Experiments

For growth rate measurements, we acclimated out of the freezer by inoculating 10 µL frozen cultures into 630 µL modified DM250 media in 48-well micotiter plates (Costar) and growing overnight on a plate shaking tower (Caliper). All growth rates were measured at the LTEE selective temperature (37°C) unless otherwise described. The modified DM media is the same as previously used throughout the evolution of these strains [5], except it contained 250 mg/L glucose and no sodium citrate. Following acclimation, saturated cultures were transferred into new plates with a 1∶64 dilution in DM media supplemented with 5 mM of a single carbon source. Under these conditions, growth in plates correlates well with growth in flasks, both with and without citrate [*p*<0.0001, R^2^ = 0.74, linear regression *F* test(1, 28) = 79.3]. The substrates analyzed were those where consistent reduction in cellular respiration were previously observed, as well as several sugars for which fitness changes had been previously measured after 2,000 generations [12] and citrate. Between 3 and 11 biological replicates were run for each strain/carbon source combination.

Optical densities were obtained every 30 min to 1 h on a Wallac Victor 2 plate reader (Perkin-Elmer), until 48 h had passed or cultures reached saturation, using a previously described automated measurement system [9],[10]. Growth rates were determined by fitting an exponential growth model using custom analysis software, Curve Fitter (N. F. Delaney, CJM, unpublished; http://www.evolvedmicrobe.com/Software.html). Representative growth curves and fitted growth rates are shown in Figure S4. The growth rate for all strains relative to the ancestor was calculated for each plate (averaging over the two ancestors, REL606 and REL607), and averaged across plates. Mean growth rates relative to the average of the ancestors were used throughout. When quantifying the number of increases and decreases in rate for evolved strains, we used all of the data for the substrates for which the ancestor exhibited growth—a necessary criterion for the evolved strains to demonstrate reductions.

### Cellular Respiration Assays

Biolog assays for respiration capacity were run as in Cooper and Lenski [4]. Briefly, cultures were grown from freezer stocks for two cycles of 1∶100 dilution and 24 h of growth in 10 mL LB in flasks shaking at 37°C. LB was used to avoid catabolite repression due to growth in minimal media, which could result in fewer positive results on nonglucose carbon sources. These cultures were inoculated 1∶100 into fresh LB and grown for 6 h before being spun down at 12,000 g for 10 min and rinsed in saline to remove residual medium. Rinsed cells were resuspended in IF-0 buffer with dye added (Biolog) to a constant density of 85% transmittance, and all wells of Biolog PM1 plates were inoculated with 100 µL of this suspension. The plates were incubated, unshaken, at 37°C. OD_600_ was measured at 0, 4, 12, 24, and 48 h, and all well readings were adjusted by subtracting the reading of the well at 0 h. A trapezoidal area approximation [4] combined the five measurements for each well into one value, which reflects the area under the curve (AUC) of optical density versus time. One replicate plate experiment was performed for each evolved strain at 20k and 50k generations, and four replicates were run for each ancestor (REL606 and REL607). Tests for tradeoffs for the evolved strains as a group on a substrate had were one-sample *t* tests against the ancestral distribution with significance cutoff of *p* = 0.002 to adjust for multiple comparisons. Tests for individual strains were the same but with a cutoff of *p* = 0.0005.

### FBA

Flux analysis was carried out with a genome-scale model of *E. coli* metabolism (iAF_1260 [58]). The model incorporates 2,382 reactions and 1,668 metabolites. The default minimal media environment and reaction bounds were used. Fluxes were predicted for each individual carbon source provided, normalized by number of carbon atoms to 10 units of glucose. Maximal biomass per substrate was used as the objective criterion as previously described [15]. To determine whether a reaction was necessary for optimal growth on a substrate, each reaction flux predicted was individually constrained to zero. Only the necessary reactions, those for which constraining the flux resulted in a reduction in final biomass, were considered in the analysis of differences between flux vectors (Table S2). Reaction differences between substrates, considered for the Hamming distance, are listed in Table S3. Table S4 summarizes alternative distance metrics that were used to assess the difference between flux vectors.

## Supporting Information

Figure S1 **Summary of parallel changes observed using Biolog assays.** Blue shading indicates catabolic function that consistently decayed across the parallel populations (statistically significant loss of function for the evolved strains as a group compared to ancestor). Red shading represents statistically significant gains of function. The number in each cell is the number of populations that significantly lost catabolic function on that carbon source relative to the ancestor (*p*<0.0005). There were 12 evolved isolates tested for all time points except 50k, for which 13 strains were tested (including A-2S). Substrates in green and italicized allowed no growth of the ancestor within 48 h in growth rate assays in DM media.
(TIFF)

Figure S2 **Respiration assay for ancestral and evolved strains on citrate.** Points show biological replicate measurements for different groups of strains. The signal for the Cit^+^ A-3 50k isolate is statistically indistinguishable from other 50k isolates.
(TIFF)

Figure S3 **Histogram showing less parallelism of metabolic declines in growth rate than respiration.** The *x* axis indicates the number of strains that exhibited a metabolic decline on a substrate. The grey bars are observed metabolic decreases, the black line is the mean observed number of decreases, and the red outline is the null distribution for a single observation given random increases and decreases. Growth rate changes for 20k (A) and 50k (B) isolates did not show the same degree of parallelism as cellular respiration declines at 20k (C) and 50k (D). The substrates considered were all those for which growth rate and respiration data were both available and for which the ancestor exhibited growth or respiration necessary for the evolved strains to demonstrate reductions. These substrates were acetate, D-alanine, D-saccharic acid, D-serine, D-sorbitol, galactose, L-alanine, L-proline, L-serine, lactate, lactose, maltose, mannose, melibiose, mono-methyl succinate, mucic acid, ribose, and trehalose.
(TIFF)

Figure S4 **Representative growth curves and fitted growth rates.** (A) Measured growth curves for 50k isolates of A-2L (red), A-1 (black). (B) Fitted growth rates from the measured growth curves.
(TIFF)

Table S1 **List of LTEE isolates used.**
(XLSX)

Table S2 **Reactions predicted by FBA as necessary for optimal growth on substrates.** These are the reactions used to compare substrate differences with FBA. The substrates for which the reactions are necessary are listed, as well as the percent of optimal growth that is possible without the reactions and related genes.
(XLSX)

Table S3 **Reactions that are not predicted to be shared between the metabolism of glucose and alternative substrates, determining the Hamming distance between substrates for FBA comparisons.**
(XLSX)

Table S4 **Summary of alternative metrics used to assess the difference between substrate utilization using FBA predictions.**
(XLSX)

## Abbreviations

20k: 20,000 generations
50k: 50,000 generations
FBA: flux balance analysis
LTEE: Lenski Long-Term Evolution Experiment

## References

[1] Law R (1979) Optimal life histories under age-specific predation. Am Nat 114: 399–417.

[2] MacArthur R (1972) Geographical ecology. Princeton, NJ: Princeton University Press.

[3] Sniegowski PD, Gerrish PJ, Lenski RE (1997) Evolution of high mutation rates in experimental populations of *E. coli* . Nature 387: 703–705. 9192894 10.1038/42701

[4] Cooper VS, Lenski RE (2000) The population genetics of ecological specialization in evolving *Escherichia coli* populations. Nature 407: 736–739 doi:10.1038/35037572 11048718 10.1038/35037572

[5] Lenski RE, Rose MR, Simpson SC, Tadler SC (1991) Long-term experimental evolution in *Escherichia coli*. I. Adaptation and divergence during 2,000 generations. Am Nat 138: 1315–1341 doi:10.1086/285289

[6] Bochner BR, Gadzinski P, Panomitros E (2001) Phenotype microarrays for high-throughput phenotypic testing and assay of gene function. Genome Res 11: 1246–1255 doi:10.1101/gr.186501 11435407 10.1101/gr.186501 PMC311101

[7] Blount ZD, Borland CZ, Lenski RE (2008) Historical contingency and the evolution of a key innovation in an experimental population of *Escherichia coli* . Proc Natl Acad Sci USA 105: 7899–7906 doi:10.1073/pnas.0803151105 18524956 10.1073/pnas.0803151105 PMC2430337

[8] Novak M, Pfeiffer T, Lenski RE, Sauer U, Bonhoeffer S (2006) Experimental tests for an evolutionary trade-off between growth rate and yield in *E. coli* . Am Nat 168: 242–251 doi:10.1086/506527 16874633 10.1086/506527

[9] Delaney NF, Rojas Echenique JI, Marx CJ (2013) Clarity: an open-source manager for laboratory automation. J Lab Autom 18: 171–177 doi:10.1177/2211068212460237 23032169 10.1177/2211068212460237 PMC5170844

[10] Delaney NF, Kaczmarek ME, Ward LM, Swanson PK, Lee M-C, et al (2013) Development of an optimized medium, strain and high-throughput culturing methods for *Methylobacterium extorquens* . PloS ONE 8: e62957 doi:10.1371/journal.pone.0062957.s006 23646164 10.1371/journal.pone.0062957 PMC3639900

[11] Vasi F, Travisano M, Lenski RE (1994) Long-term experimental evolution in *Escherichia coli*. II. Changes in life-history traits during adaptation to a seasonal environment. Am Nat 144 (3) 432–456.

[12] Travisano M, Lenski RE (1996) Long-term experimental evolution in *Escherichia coli*. IV. Targets of selection and the specificity of adaptation. Genetics 143: 15–26. 8722758 10.1093/genetics/143.1.15 PMC1207249

[13] Leiby N, Harcombe WR, Marx CJ (2012) Multiple long-term, experimentally-evolved populations of *Escherichia coli* acquire dependence upon citrate as an iron chelator for optimal growth on glucose. BMC Evol Biol 12: 151 doi:10.1186/1471-2148-12-151 22909317 10.1186/1471-2148-12-151 PMC3496695

[14] Orth JD, Thiele I, Palsson BO (2010) What is flux balance analysis? Nat Biotechnol 28: 245–248 doi:10.1038/nbt.1614 20212490 10.1038/nbt.1614 PMC3108565

[15] Harcombe WR, Delaney NF, Leiby N, Klitgord N, Marx CJ (2013) The ability of flux balance analysis to predict evolution of central metabolism scales with the initial distance to the optimum. PLoS Comput Biol 9: e1003091 doi:10.1371/journal.pcbi.1003091 23818838 10.1371/journal.pcbi.1003091 PMC3688462

[16] Wielgoss S, Barrick JE, Tenaillon O, Wiser MJ, Dittmar WJ, et al (2013) Mutation rate dynamics in a bacterial population reflect tension between adaptation and genetic load. Proc Natl Acad Sci USA 110: 222–227 doi:10.1073/pnas.1219574110 23248287 10.1073/pnas.1219574110 PMC3538217

[17] Barrick JE, Yu DS, Yoon SH, Jeong H, Oh TK (2009) Genome evolution and adaptation in a long-term experiment with *Escherichia coli* . Nature 461: 1243–1247 doi:10.1038/nature08480 -sup 19838166 10.1038/nature08480

[18] Bloom JD, Silberg JJ, Wilke CO, Drummond DA, Adami C, et al (2005) Thermodynamic prediction of protein neutrality. Proc Natl Acad Sci U S A 102: 606–611. 15644440 10.1073/pnas.0406744102 PMC545518

[19] Tokuriki N, Stricher F, Schymkowitz J, Serrano L, Tawfik DS (2007) The stability effects of protein mutations appear to be universally distributed. J Mol Biol 369: 1318–1332 doi:10.1016/j.jmb.2007.03.069 17482644 10.1016/j.jmb.2007.03.069

[20] DePristo MA, Weinreich DM, Hartl DL (2005) Missense meanderings in sequence space: a biophysical view of protein evolution. Nat Rev Genet 6: 678–687 doi:10.1038/nrg1672 16074985 10.1038/nrg1672

[21] Cooper VS, Schneider D, Blot M, Lenski RE (2001) Mechanisms causing rapid and parallel losses of ribose catabolism in evolving populations of *Escherichia coli* B. J Bacteriol 183: 2834–2841 doi:10.1128/JB.183.9.2834 11292803 10.1128/JB.183.9.2834-2841.2001 PMC99500

[22] Meyer JR, Agrawal AA, Quick RT, Dobias DT, Schneider D, et al (2010) Parallel changes in host resistance to viral infection during 45,000 generations of relaxed selection. Evolution 64: 3024–3034 doi:10.1111/j.1558-5646.2010.01049.x 20550574 10.1111/j.1558-5646.2010.01049.x

[23] Travisano M, Vasi F, Lenski RE (1995) Long-term experimental evolution in *Escherichia coli*. III. Variation among replicate populations in correlated responses to novel environments. Evolution 49: 189–200. 10.1111/j.1558-5646.1995.tb05970.x 28593661

[24] Pelosi L, Kühn L, Guetta D, Garin J, Geiselmann J, et al (2006) Parallel changes in global protein profiles during long-term experimental evolution in Escherichia coli. Genetics 173: 1851–1869 doi:10.1534/genetics.105.049619 16702438 10.1534/genetics.105.049619 PMC1569701

[25] Remold S (2012) Understanding specialism when the jack of all trades can be the master of all. Proc Biol Sci 279: 4861–4869 doi:10.1371/journal.pcbi.1000112 23097515 10.1098/rspb.2012.1990 PMC3497242

[26] Burch CL, Chao L (2004) Epistasis and its relationship to canalization in the RNA virus phi 6. Genetics 167: 559–567 doi:10.1534/genetics.103.021196 15238511 10.1534/genetics.103.021196 PMC1470902

[27] Remold SK, Lenski RE (2004) Pervasive joint influence of epistasis and plasticity on mutational effects in *Escherichia coli* . Nat Genet 36: 423–426 doi:10.1038/ng1324 15072075 10.1038/ng1324

[28] Flynn KM, Cooper TF, Moore FBG, Cooper VS (2013) The environment affects epistatic interactions to alter the topology of an empirical fitness landscape. PLoS Genet 9: e1003426 doi:10.1371/journal.pgen.1003426 23593024 10.1371/journal.pgen.1003426 PMC3616912

[29] Carroll SM, Lee M-C, Marx CJ (in press) Sign epistasis limits evolutionary tradeoffs at the confluence of single- and multi-carbon metabolism in *Methylobacterium extorquens* AM1. Evolution 10.1111/evo.12301 24164359

[30] Cooper TF, Ostrowski EA, Travisano M (2007) A negative relationship between mutation pleiotropy and fitness effect in yeast. Evolution 61: 1495–1499 doi:10.1111/j.1558-5646.2007.00109.x 17542856 10.1111/j.1558-5646.2007.00109.x

[31] Khan AI, Dinh DM, Schneider D, Lenski RE, Cooper TF (2011) Negative epistasis between beneficial mutations in an evolving bacterial population. Science 332: 1193 doi:10.1126/science.1203801 21636772 10.1126/science.1203801

[32] Ostrowski EA, Rozen DE, Lenski RE (2005) Pleiotropic effects of beneficial mutations in *Escherichia coli* . Evolution 59: 2343–2352. 16396175

[33] Lee M-C, Chou H-H, Marx CJ (2009) Asymmetric, bimodal trade-offs during adaptation of *Methylobacterium* to distinct growth substrates. Evolution 63: 2816–2830 doi:10.1111/j.1558-5646.2009.00757.x 19545267 10.1111/j.1558-5646.2009.00757.x

[34] Chou H-H, Berthet J, Marx CJ (2009) Fast growth increases the selective advantage of a mutation arising recurrently during evolution under metal limitation. PLoS Genet 5: e1000652 doi:10.1371/journal.pgen.1000652 19763169 10.1371/journal.pgen.1000652 PMC2732905

[35] Cooper TF, Rozen DE, Lenski RE (2003) Parallel changes in gene expression after 20,000 generations of evolution in *Escherichia coli* . Proc Natl Acad Sci U S A 100: 1072–1077. 12538876 10.1073/pnas.0334340100 PMC298728

[36] Lee M-C, Marx CJ (2012) Repeated, selection-driven genome reduction of accessory genes in experimental populations. PLoS Genet 8: e1002651 doi:10.1371/journal.pgen.1002651 22589730 10.1371/journal.pgen.1002651 PMC3349727

[37] Le Gac M, Plucain J, Hindré T, Lenski RE, Schneider D (2012) Ecological and evolutionary dynamics of coexisting lineages during a long-term experiment with *Escherichia coli* . Proc Natl Acad Sci USA 109: 9487–9492 doi:10.1073/pnas.1207091109 22645336 10.1073/pnas.1207091109 PMC3386082

[38] Rozen DE, Philippe N, Arjan de Visser J, Lenski RE, Schneider D (2009) Death and cannibalism in a seasonal environment facilitate bacterial coexistence. Ecol Lett 12: 34–44 doi:10.1111/j.1461-0248.2008.01257.x 19019196 10.1111/j.1461-0248.2008.01257.x

[39] Rozen DE, Lenski RE (2000) Long-term experimental evolution in *Escherichia coli*. VIII. Dynamics of a balanced polymorphism. Am Nat 155: 24–35 doi:10.1086/303299 10657174 10.1086/303299

[40] de Visser JAGM, Lenski RE (2002) Long-term experimental evolution in *Escherichia coli*. XI. Rejection of non-transitive interactions as cause of declining rate of adaptation. BMC Evol Biol 2: 19. 12443537 10.1186/1471-2148-2-19 PMC134600

[41] Blount ZD, Barrick JE, Davidson CJ, Lenski RE (2012) Genomic analysis of a key innovation in an experimental *Escherichia coli* population. Nature 489: 513–518 doi:10.1038/nature11514 22992527 10.1038/nature11514 PMC3461117

[42] Barve A, Wagner A (2013) A latent capacity for evolutionary innovation through exaptation in metabolic systems. Nature 500: 203–206 doi:10.1038/nature12301 23851393 10.1038/nature12301

[43] Woods R, Schneider D, Winkworth CL, Riley MA, Lenski RE (2006) Tests of parallel molecular evolution in a long-term experiment with Escherichia coli. Proc Natl Acad Sci U S A 103: 9107–9112 doi:10.1073/pnas.0602917103 16751270 10.1073/pnas.0602917103 PMC1482574

[44] Barton NH (2000) Genetic hitchhiking. Philos Trans R Soc Lond, B, Biol Sci 355: 1553–1562 doi:10.1098/rstb.2000.0716 11127900 10.1098/rstb.2000.0716 PMC1692896

[45] Smith JM, Haigh J (1974) The hitch-hiking effect of a favourable gene. Genet Res 23: 23–35 doi:10.1017/S0016672300014634 4407212

[46] Funchain P, Yeung A, Stewart JL, Lin R, Slupska MM, et al (2000) The consequences of growth of a mutator strain of Escherichia coli as measured by loss of function among multiple gene targets and loss of fitness. Genetics 154: 959–970. 10757746 10.1093/genetics/154.3.959 PMC1461004

[47] Andersson DI, Hughes D (1996) Muller's ratchet decreases fitness of a DNA-based microbe. Proc Natl Acad Sci U S A 93: 906–907. 8570657 10.1073/pnas.93.2.906 PMC40156

[48] Rainey PB, Travisano M (1998) Adaptive radiation in a heterogeneous environment. Nature 32: 69–72. 9665128 10.1038/27900

[49] Rosenzweig RF, Sharp RR, Treves DS, Adams J (1994) Microbial evolution in a simple unstructured environment: genetic differentiation in *Escherichia coli* . Genetics 137: 903–917. 7982572 10.1093/genetics/137.4.903 PMC1206068

[50] Friesen ML, Saxer G, Travisano M, Doebeli M (2004) Experimental evidence for sympatric ecological diversification due to frequency-dependent competition in *Escherichia coli* . Evolution 58: 245–260. 15068343

[51] LeClerc JE, Li B, Payne WL, Cebula TA (1996) High mutation frequencies among *Escherichia coli* and *Salmonella* pathogens. Science 274: 1208–1211. 8895473 10.1126/science.274.5290.1208

[52] Chao L, Cox EC (1983) Competition between high and low mutating strains of *Escherichia coli* . Evolution 37: 125–134. 10.1111/j.1558-5646.1983.tb05521.x 28568016

[53] Giraud A, Matic I, Tenaillon O, Clara A, Radman M, et al (2001) Costs and benefits of high mutation rates: adaptive evolution of bacteria in the mouse gut. Science 291: 2606–2608 doi:10.1126/science.1056421 11283373 10.1126/science.1056421

[54] McDonald MJ, Hsieh Y-Y, Yu Y-H, Chang S-L, Leu J-Y (2012) The evolution of low mutation rates in experimental mutator populations of Saccharomyces cerevisiae. Curr Biol 22: 1235–1240 doi:10.1016/j.cub.2012.04.056 22727704 10.1016/j.cub.2012.04.056

[55] Turrientes M-C, Baquero F, Levin BR, Martínez J-L, Ripoll A, et al (2013) Normal mutation rate variants arise in a mutator (Mut S) Escherichia coli population. PloS ONE 8: e72963 doi:10.1371/journal.pone.0072963.s011 24069167 10.1371/journal.pone.0072963 PMC3771984

[56] Woese CR (1987) Bacterial evolution. Microbiol Rev 51: 221–271. 2439888 10.1128/mr.51.2.221-271.1987 PMC373105

[57] Rozen DE, Schneider D, Lenski RE (2005) Long-term experimental evolution in *Escherichia coli*. XIII. Phylogenetic history of a balanced polymorphism. J Mol Evol 61: 171–180 doi:10.1007/s00239-004-0322-2 15999245 10.1007/s00239-004-0322-2

[58] Feist AM, Henry CS, Reed JL, Krummenacker M, Joyce AR, et al (2007) A genome-scale metabolic reconstruction for *Escherichia coli* K-12 MG1655 that accounts for 1260 ORFs and thermodynamic information. Mol Syst Biol 3: 121 doi:10.1038/msb4100155 17593909 10.1038/msb4100155 PMC1911197

[59] Leiby N, Marx CJ (2013) Data from: Metabolic erosion primarily through mutation accumulation, and not tradeoffs, drives limited evolution of substrate specificity in *Escherichia coli* . Dryad Digital Repository http://dx.doi.org/10.5061/dryad.7g401. 10.1371/journal.pbio.1001789 PMC3928024 24558347

