# Supplementary figures and images for: Metabolic Erosion Primarily Through Mutation Accumulation, and Not Tradeoffs, Drives Limited Evolution of Substrate Specificity in Escherichia coli
Source: PLoS Biol. 2014 Feb 18;12(2):e1001789. doi: 10.1371/journal.pbio.1001789 (PMC3928024; doi:10.1371/journal.pbio.1001789)

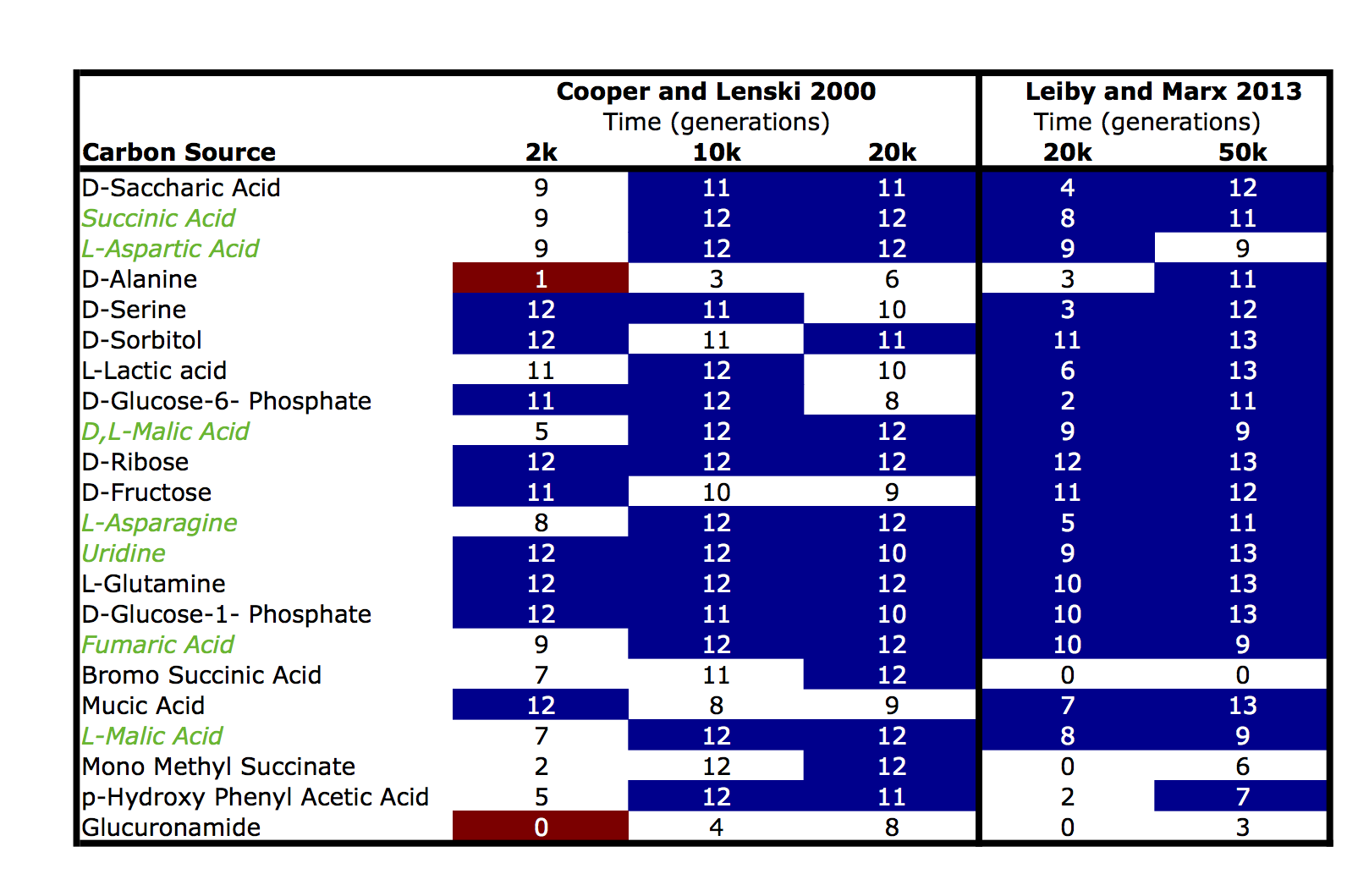

Supplement: Figure S1 — Summary of parallel changes observed using Biolog assays. Blue shading indicates catabolic function that consistently decayed across the parallel populations (statistically significant loss of function for the evolved strains as a group compared to ancestor). Red shading represents statistically significant gains of function. The number in each cell is the number of populations that significantly lost catabolic function on that carbon source relative to the ancestor (p<0.0005). There were 12 evolved isolates tested for all time points except 50k, for which 13 strains were tested (including A-2S). Substrates in green and italicized allowed no growth of the ancestor within 48 h in growth rate assays in DM media. (TIFF) [file pbio.1001789.s001.tif]

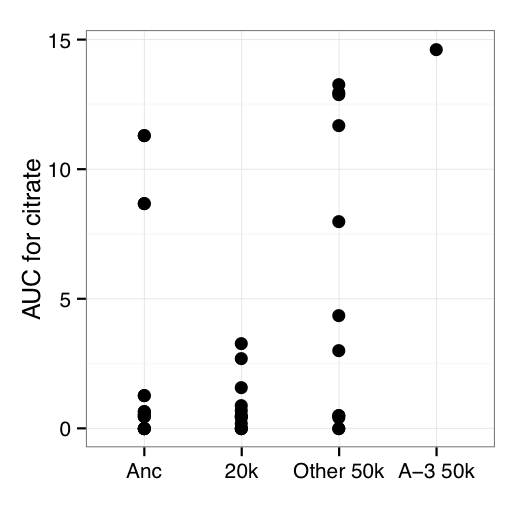

Supplement: Figure S2 — Respiration assay for ancestral and evolved strains on citrate. Points show biological replicate measurements for different groups of strains. The signal for the Cit+ A-3 50k isolate is statistically indistinguishable from other 50k isolates. (TIFF) [file pbio.1001789.s002.tif]

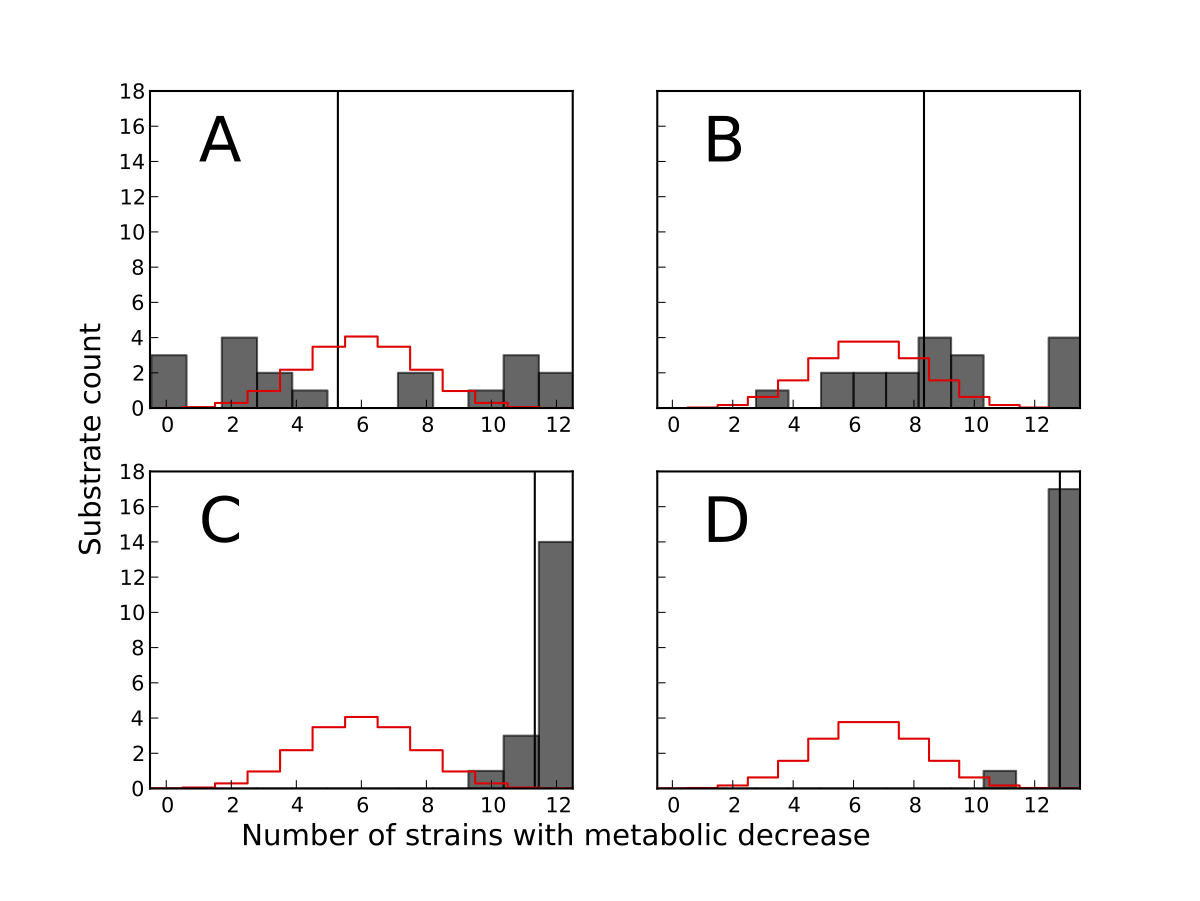

Supplement: Figure S3 — Histogram showing less parallelism of metabolic declines in growth rate than respiration. The x axis indicates the number of strains that exhibited a metabolic decline on a substrate. The grey bars are observed metabolic decreases, the black line is the mean observed number of decreases, and the red outline is the null distribution for a single observation given random increases and decreases. Growth rate changes for 20k (A) and 50k (B) isolates did not show the same degree of parallelism as cellular respiration declines at 20k (C) and 50k (D). The substrates considered were all those for which growth rate and respiration data were both available and for which the ancestor exhibited growth or respiration necessary for the evolved strains to demonstrate reductions. These substrates were acetate, D-alanine, D-saccharic acid, D-serine, D-sorbitol, galactose, L-alanine, L-proline, L-serine, lactate, lactose, maltose, mannose, melibiose, mono-methyl succinate, mucic acid, ribose, and trehalose. (TIFF) [file pbio.1001789.s003.tif]

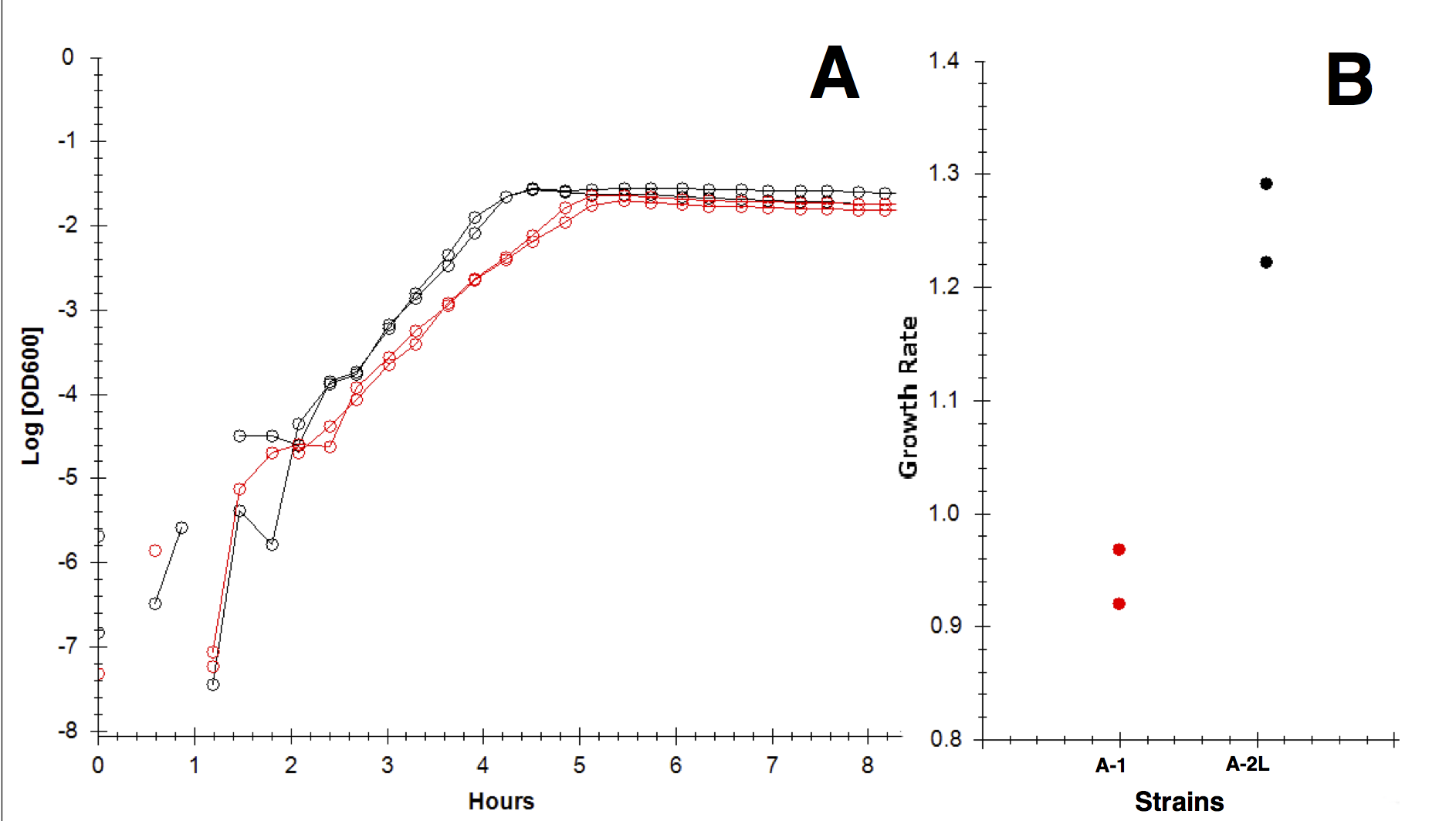

Supplement: Figure S4 — Representative growth curves and fitted growth rates. (A) Measured growth curves for 50k isolates of A-2L (red), A-1 (black). (B) Fitted growth rates from the measured growth curves. (TIFF) [file pbio.1001789.s004.tif]
